# Everything, everywhere, all at once: A study of polychronicity, work-school facilitation, and emotional exhaustion in working students

**DOI:** 10.3389/fpsyg.2023.976874

**Published:** 2023-02-06

**Authors:** Allan Grogan, Juliana Lilly

**Affiliations:** Department of Management, Marketing, and Information Systems, Sam Houston State University, Huntsville, TX, United States

**Keywords:** polychronicity, work-school facilitation, emotional exhaustion, mediation analysis, working students, conservation of resources theory

## Abstract

This study investigates how a preference for multitasking serves as a resource toward mitigating emotional exhaustion through the mediating role of work-school facilitation. Utilizing conservation of resources (COR) theory, we utilized a sample of 153 working students in a time-lagged study to examine the relationship between these variables. Results suggest that polychronicity serves as a valuable resource toward balancing the simultaneous needs of work and school, and ultimately, decreasing academic emotional exhaustion. The findings presented in this study further advance scholarship in this domain by highlighting our understanding of polychronicity as an individual difference variable in facilitating role balance within the school–work domain. Practical implications and limitations are discussed.

## Introduction

A recent study by the National Center for Education Statistics (2018) found that over 40 percent of full-time students in the United States were employed 40 hours per week or more (National Center for Education Statistics, 2018). This trend toward balancing the simultaneous needs of work and school is exacerbated by the prevalence of technology making almost everything, everywhere to be available at the touch of a button. Determining which information is valid, how to use it, and how to go about one’s daily tasks and routines using this information can be challenging. College students who also work face unique challenges as they navigate multiple obligations while being exposed to massive amounts of information from school, work, family, friends, and media, possibly leading to increased stress and anxiety in all facets of their life, but particularly at school since the cost of school failure is high.

When working students fail on the job, they often have other opportunities for employment. However, when they fail at school, the costs are often significant in terms of their financial costs, with the average student debt approaching $26,000 for public, 4-year bachelor degree institutions (National Center for Education Statistics, 2020). Moreover, recent research investigating stress in working students found this segment of the population experienced higher burnout in school-related situations than in work-related situations (Draghici and Cazan, 2022). Since stress resulting from multiple sources may lead to increased levels of academic emotional exhaustion (Kremer, 2016; McNall and Michel, 2017), which is characterized which is defined as being emotionally overextended and exhausted by one’s studies, researchers have shown an increasing interest in multitasking as it relates to balancing the needs of work and school. The current study investigates how one’s orientation toward multitasking, also referred to as polychronicity, will mitigate emotional exhaustion in the school domain for a sample of working students.

Based on early scholarship by Hall (1959), individuals differ on how they perceive, prioritize, and allocate their time. Those individuals who prefer to undertake and manage multiple activities at a time have a polychronic orientation toward time while individuals who prefer to complete one task prior to embarking on another are said to have a monochromic orientation (Bluedorn et al., 1992). Polychronicity is distinct from other personality traits (Conte and Jacobs, 2003) and the increased flexibility associated with one’s orientation toward multitasking makes this an ideal individual difference variable to investigate its influence on emotional exhaustion in this segment of the population since the way work is performed may influence one’s psychological responses to that work.

Prior empirical research has found that polychronicity negatively correlates to turnover intentions (Jang and George, 2012) and is positively related to job performance (Asghar et al., 2021), job satisfaction, and work engagement (Conte et al., 2019). Polychronicity was also found to reduce role ambiguity (Fournier et al., 2013). Moreover, recent scholarship investigating work–family conflict found that polychronicity serves as a negative antecedent to work overload, thus suggesting that a preference for undertaking multiple roles serves as an important resource for mitigating stress (Korabik et al., 2017). These findings suggest that an orientation toward multitasking should serve as a resource toward balancing simultaneous work and school responsibilities among working students. Indeed, the vast majority of empirical studies appearing in the work-life and work-school domains have focused on conflict and stress from undertaking multiple roles (Greenhaus and Powell, 2006; Choo et al., 2021) rather than investigating their positive aspects.

Conservation of resources (COR) theory is used to investigate the relationship between polychronicity and emotional exhaustion. COR theory, as postulated by Hobfoll et al. (2018), states that individuals are motivated to acquire, protect, and retain resources which they value, as well as acquire new resources to help them respond to stressful conditions such as anxiety, depression, and work–family conflict. In this regard, resources are characterized as states, objects, or conditions of value. More specifically, resources can be considered as anything perceived by an individual to help attain their goal (Halbesleben et al., 2014), and can be classified as organizational or personal in nature (Schaufeli, 2017). These types of personal or psychological resources include malleable personal attributes, such as self-esteem, optimism, and self-efficacy, or stable, individual difference traits, such as extraversion, emotional stability, and locus of control (Halbesleben et al., 2014). As such, we consider polychronicity to be a resource as well. Emotional exhaustion, viewed through the lens of COR theory, is associated with the loss of key resources such as leisure and rest when students experience stress from undertaking their work and school roles simultaneously. We contend that working students utilize their orientation for multitasking to not only preserve their resources, but to also negotiate the simultaneous demands of work and school in efforts to prevent emotional exhaustion in their studies.

We introduce an additional theoretical framework, work-family enrichment theory (Greenhaus and Powell, 2006) to account for the transfer of resources across roles. This theory, which contends that resources obtained in one role such as work, may facilitate or enrich another role such as family, school, or well-being. Consistent with this theory, we utilize work-school facilitation, which is defined as “improvement in the quality of the school role resulting from participation in work” (Butler, 2007, p. 501), as a mediator to account for the polychronicity-emotional exhaustion relationship. Work-school facilitation has been shown to increase student dedication and well-being (Creed et al., 2015), life satisfaction (Cinamon, 2016), and performance at school (Butler, 2007). We contend that polychronicity, an individual difference trait, will enable working students to balance the competing needs of work and school through increased enrichment in their work and school roles. The enrichment gained by working students is expected to spill over into the school domain, thus decreasing their emotional exhaustion.

This study makes two important contributions to existing scholarship. First, we investigate how individual differences in working students’ orientation for multitasking (polychronicity) will influence their emotional exhaustion toward school. Prior empirical literature has primarily investigated polychronicity within the workplace with few studies examining the effect of this trait in the academic context (Capdeferro et al., 2014). Our second contribution expands scholarship into the work-school interface by examining how polychronicity, considered a stable personality trait (Slocombe and Bluedorn, 1999), serves as a resource for working students to enrich their work and school roles, by extending the work-family theoretical framework of Greenhaus and Powell (2006) into the work-school domain. In doing so, we answer the call by Nicklin and McNall (2013) for empirical research to consider other psychological traits such as polychronicity in the work-school context. Moreover, we are not aware of any empirical study investigating polychronicity as an antecedent to work-school facilitation in this domain.

## Theory and hypotheses

According to COR theory, resources may encompass numerous social, physical, or psychological attributes perceived by an individual to help them attain their goals or objectives (Halbesleben et al., 2014). Moreover, individuals with an accumulation of resources are in a better position to resist stressful circumstances that may adversely impact their well-being (Hobfoll et al., 2018). Halbesleben et al. (2014) identified numerous personality factors and traits, which serve as positive psychological resource. Previously investigated within COR theory, empirical studies investigating these “key” traits include self-efficacy and self-esteem (Xanthopoulou et al., 2009), conscientiousness (Halbesleben et al., 2009), and core-self-evaluations (McNall and Michel, 2011). Specific to the current study, working students with greater resources who experience the daily demands from their work and their studies are more capable of avoiding negative outcomes, such as stress, anxiety, and emotional exhaustion.

Another proposition of COR theory states that those individuals who have a greater reservoir of resources have a greater opportunity to invest these resources (Halbesleben et al., 2014). In this regard, polychronicity may be considered a buffer to protect against the threat of resource loss. Being a stable personality trait, polychronicity aims to increase working students’ ability to undertake multiple roles, providing them with the ability to further increase and invest their resources in efforts to prevent exhaustion. Korabik et al. (2017) found polychronicity was negatively related to workload, with lower workload being related to lower turnover intentions and higher family satisfaction, and ultimately life satisfaction. In a similar fashion, Mittal and Bienstock (2018) proposed a direct positive association between polychronicity and life satisfaction based on their orientation to multitask and lower conflict among different their work and life roles. In particular, Mittal and Bienstock (2018) note that polychronicity serves as a key personal resource by “…allowing people to manage their time more effectively, thereby lowering the level of overall conflict” (Mittal and Bienstock, 2018, p. 464). Extending this rationale to the work-school interface, polychronicity is expected to serve as a key personal resource for working students not only by enriching their experiences at work and school, but also through mitigating their feelings of exhaustion in their studies.

As previously mentioned, conservation of resources theory states that resources are integral to enabling an individual to achieve their goals (Halbesleben et al., 2014). We contend that polychronic students will find it easier to multitask on and accomplish their daily responsibilities, tasks, and projects, and apply these skills and experiences gained in the workplace to their school roles. We contend that the value added resource of polychronicity is best summarized by Kaufman et al. (1991) who note that these individuals “…are likely to combine some of their cross-role demands into efficient periods of polychronic time use, neatly dovetailing several demands through the complementary use of their time, skills, and energy resources” (p. 394). Providing a contextual example of facilitation would entail being able to alternate between helping customers and performing administrative tasks at work. These enriching experiences gained on the job would further enable working students to mitigate exhaustion by meeting academic deadlines, performing well on exams, and succeeding in their studies. Therefore, we expect working students with a polychronic orientation to enrich, or facilitate their work and school roles, thus leading to our first hypothesis:

*H1*: Polychronicity is positively related to work-school facilitation.

Enrichment theory (Sieber, 1974; Greenhaus and Powell, 2006) contends that participating in certain roles creates resources, which can benefit and transfer to other roles. Working students who experience positive affect from the experiences, skills, social capital, and increased flexibility associated with work-school facilitation will experience low levels of emotional exhaustion in their role at school. Prior scholarship in the work-family domain has found support for both direct enhancement in the originating domain and cross-transfer of resources into other roles (Carlson et al., 2014; McNall et al., 2021). Extending enrichment theory into the academic context, Butler (2007) found work-school enrichment associated with increased school satisfaction and performance. In addition, McNall and Michel (2017) found that work-school enrichment was positively related to general psychological health and negatively related to burnout (McNall and Michel, 2017), suggesting that the positive experiences and skills working students obtain on the job benefit, or spill over to their role in university. Following this logic, we expect work-school facilitation to have a positive impact on working students and mitigate exhaustion in their studies, advancing the following hypothesis:

*H2*: Work-school facilitation is negatively related to emotional exhaustion.

Prior empirical research investigating polychronicity has yielded mixed findings on various academic and workplace outcomes and on inter-role conflict (König and Waller, 2010; Korabik et al., 2017). One reason for this negative relationship can be attributed to the fragmented attention span of polychrons, which prevents them from gaining a deeper focus and understanding of the tasks at hand, leading to cognitive exhaustion (Madjar and Shalley, 2008). Another reason may be explained by their lack of punctuality and being able to meet deadlines (Bluedorn et al., 1999; Conte and Jacobs, 2003). Conversely, the preference polychrons exhibit toward completing simultaneous tasks makes them less prone to stress from role overload (Kaufman et al., 1991) while increasing their satisfaction on the job (Hecht and Allen, 2005; Arndt et al., 2006). This increased enrichment through the skills and experiences gained on the job is integral to reducing stress and exhaustion at school. In addition, Schaufeli (2017) argues that stable personality traits such as extraversion and emotional stability will serve as antecedents to reducing the stress from job demands. Building upon Hypotheses 1 and 2, we integrate a mediational framework with polychronicity as a distal, individual difference variable increasing work-school facilitation, a proximal variable, integral to reducing emotional exhaustion. Given that polychronicity is not expected to have an effect on emotional exhaustion directly, we rely on work-school facilitation to mediate this proposed framework. Based on this rationale, we propose the following hypothesis:

*H3*: Work-school facilitation mediates the relationship between polychronicity and emotional exhaustion.

## Methods

### Participants

We collected surveys from 153 third and fourth-year students enrolled in an undergraduate management course from a medium-sized public university in Texas. Students included in this sample were registered either in online or face-to-face sections of their courses and worked an average of 32 h per week. This sample included 71% females, 28% males, and 1% who declined to answer, with an average age of 25 years old. Sample respondents identified as the following: African American: 11%, Hispanic: 35%, Caucasian: 40%, Asian: 5%, Native American: 1%, Middle Eastern: 1%, and other ethnicity: 3%, while 5% of respondents chose not to answer. Students who voluntarily participated were provided extra credit, which counted toward their final grade in the course.

### Design

We implemented a time lag of 4 weeks between collecting data for our independent variables of polychronicity and work-school facilitation, and emotional exhaustion to mitigate against common method bias (Podsakoff et al., 2003). Students were provided an online survey link in Qualtrics for Waves 1 and 2 of the survey.

### Measures

#### Polychronicity

We assessed polychronicity using the Inventory of Polychronic Values (IPV, Bluedorn et al., 1999). This 10-item scale was modified to measure this concept at the individual level by replacing each item stem from “We” to “I.” A sample item included “I like to juggle several activities at the same time.” Responses were recorded on a seven-point Likert scale with values ranging from 1 “strongly disagree” to 7 “strongly agree” (α = 0.81).

#### Work-school facilitation

Work-school facilitation was measured using five items from Butler (2007). Sample items included “The skills I use on my job are useful for things that I have to do at school.” Responses were recorded on a five-point Likert scale with values ranging from 1 “never” to “very often” (α = 0.82).

#### Emotional exhaustion

We measured emotional exhaustion from work using a seven-item scale (Maslach and Jackson, 1981), adapted for the academic context (*cf.* McNall and Michel, 2017). Participants were asked to rate their frequency with being exhausted from their studies ranging from 1 = strongly disagree to 7 = strongly agree. A sample item included “I feel emotionally drained from my school” (α = 0.90).

### Analysis

We employed Hayes’ PROCESS methodology (Model #4) using a macro for SPSS (Hayes, 2013) utilizing 5,000 bootstrapping iterations. All effects are considered significant if their bias-corrected confidence intervals excluded zero. The level of significance was 0.05 for all regressions performed in this study.

### Statistical control

We controlled for job and school-oriented factors to ensure that variation in emotional exhaustion was not being driven by demands associated with these contexts. In accordance with Wyland et al. (2013), we controlled for the number of credit hours per semester with an indicator for the following categories: 1–3; 4–6; 7–9; and 12 plus credit hours. In addition, we controlled for the number hours worked per week and the participant’s position within the organization with an indicator variable for the following categories: non-management; front-line management; and middle or upper management. Finally, to mitigate the possibility that emotional exhaustion is attributed to whether the student is enrolled in online or in-person learning, we included an indicator variable if the student was registered in an online course.

## Results

**Figure 1:**
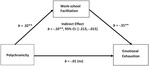
Mediation model outlining the relationship between polychronicity and emotional exhaustion. ^**^p < 0.05.

Table 1 reports the correlations, respective means, standard deviations, and reliabilities for all variables used in the study. Figure 1 illustrates the paths of our hypothesized model. As expected, we did not find a direct effect between polycnronicity and emotional exhaustion. Consistent with Hypothesis 1 and 2 respectively, we found that polychronicity was positively related to work-school facilitation (*point estimate* = 0.32, *SE* = 0.08, *CI_95%_* = 0.157, 0.486) and work-school facilitation was negatively related to emotional exhaustion (*point estimate* = −0.31, *SE* = −0.13, *CI_95%_* = −0.559, −0.056). We found no evidence of a direct effect between polychronicity and emotional exhaustion (*point estimate* = −0.01, *SE* = 0.13, *CI_95%_* = −0.281, 0.253); however, we found a negative, and significant indirect effect for the relationship between polychronicity and emotional exhaustion when mediated by work-school facilitation (*point estimate* = −0.10, *SE* = −0.05, *CI_95%_* = −0.213, −0.016), thus supporting Hypothesis 3. As an additional measure of robustness, we ran a separate analysis in PROCESS setting the level of significance to 0.01 and our effect sizes remain unchanged. When introducing control variables in our study, our effect sizes remained similar, however their level of significance decreased to 0.05.

**Table 1:** Descriptive statistics and correlation matrix.

	M	SD	1	2	3	4	5
Polychronicity	3.58	0.94	(0.81)				
Work-school Facilitation	3.17	0.98	0.29^**^	(0.82)			
Emotional Exhaustion	3.46	1.47	−0.05	−0.23^**^	(0.90)		
Credit Hours	3.58	0.80	0.08	−0.30^**^	0.07		
Work Hours	31.89	12.55	0.09	0.18^*^	−0.18^*^	−0.33^**^	
Position in Company	1.35	0.64	0.08	0.19^*^	0.02	−0.16^*^	0.39^**^

*N* = 153. Credit hours were measured with an indicator variable with the following categories: 1–3; 4–6; 7–9; and 12 plus credits. Position was measured with an indicator variable with the following categories: non-management; front-line management; and middle or top management. Reliabilities indicated in parenthesis.

^*^p < 0.05; ^**^p < 0.01.

## Discussion

The current study investigated how polychronicity in working students serves as a resource to mitigate emotional exhaustion through the mediating mechanism of work-school facilitation. This orientation for engaging in multiple tasks simultaneously led to an increase in work-school facilitation, which subsequently decreased exhaustion. Our findings remained consistent when controlling for the number of credits enrolled, hours worked per week and position within the organization.

### Theoretical implications

The findings from this study are consistent with prior scholarship which found polychronicity associated with greater work engagement (Conte et al., 2019) as well as higher levels of positive affect and self-efficacy, in addition to lower levels of psychological strain (Hecht and Allen, 2005). Our results suggest that polychronicity serves as a resource by enabling those with a time management orientation toward multitasking to more effectively balance multiple tasks and responsibilities to their advantage, thus mitigating school-related exhaustion. In doing so, we provided a unique contribution to the existing scholarship in polychronicity by investigating this individual difference trait in working students. Borrowing from the work-life literature, we integrated a mediational framework within the school-work interface to investigate this under-researched domain (Choo et al., 2021). This is the first study, to the best of our knowledge, to jointly investigate work-school facilitation as a mediator to account for the relationship between polychronicity and emotional exhaustion.

Consistent with the central premise of COR theory (Hobfoll et al., 2018), our findings underscore the importance of personal resources in counteracting stressful situations by balancing the needs of work-school obligations. By examining how individual differences in student multitasking orientations can mitigate exhaustion, we expand the use of stable personality traits within COR theory. In doing so, we expand upon recent scholarship in the work-family domain literature (Korabik et al., 2017), by investigating polychronicity within the work-school context.

### Limitations

We acknowledge some limitations inherent to our study. Our sample investigated work-school facilitation and emotional exhaustion in undergraduate working students, and therefore, our findings may not generalize to other academic majors or graduate student populations. Rather than identifying themselves as working students (*cf.* Butler, 2007), graduate students, in particular those pursuing an MBA, may consider themselves as “employees who are enrolled in courses” (Wyland et al., 2013, p. 348). In this scenario, these individuals may be in a better position to negotiate the competing demands of both work and school, irrespective of their preference to multitask. We acknowledge another limitation in the current study is the sample size, which may not be representative of the large percentage of students across majors outside of business school students, thus limiting the external validity of our study. Finally, our model only investigated polychronicity as a resource toward mitigating emotional exhaustion. It is plausible that other resources of a personal (self-efficacy and locus of control) and contextual orientation (supervisor and coworker support) may serve to increase work-school facilitation and reduce emotional exhaustion in working students. Future research investigating a combination of these personal resources holds great promise.

### Practical implications

Our findings may also provide a partial explanation for the current labor shortage. The “Great Resignation” occurring in the past few years has created a problem for organizations struggling to fill openings, and shortages in customer service industries such as retail and hospitality are particularly severe (Goldberg, 2022). These jobs are often filled by college students due to the flexible nature of the work, which allows students to work while going to school. However, students who have difficulty navigating multiple obligations due to having access to everything, everywhere, all at once may experience increased stress and anxiety and, as a result, stop working. Indeed, traditional college students today are part of Generation Z, the generation most likely to experience stress according to researchers (American Psychological Association, 2020). If students learn how to develop their ability to multitask, these student employees may be less prone to emotional exhaustion; thus improving their mental health, improving their academic progress, and providing economic benefits to themselves and society. Finally, although we did not directly investigate the effects of polychronicity in the workplace, prior research has found this concept associated with lower levels of work overload (Korabik et al., 2017) as well as increased job satisfaction and work engagement (Conte et al., 2019). In this regard, managers may wish to identify those working students with an orientation toward multitasking in efforts to enrich their work experiences, and ultimately their job satisfaction (Wyland et al., 2016).

## Conclusion

This study contributed to the literature in school and work balance by investigating how polychronicity serves as a resource for mitigating emotional exhaustion. Our findings underscore the importance of integrating mediation analysis to further determine how individual differences in time orientation influence personal outcomes. We hope that our findings guide future scholarship to further investigate the interface between role conflict and facilitation within this domain.

## Data availability statement

The raw data supporting the conclusions of this article will be made available by the authors, without undue reservation.

## Ethics statement

The studies involving human participants were reviewed and approved by this study was approved by the Office of Research and Sponsored Programs at Sam Houston State University (IRB-2021-287) prior to collection of data. All data were collected in accordance with standard ethical guidelines governing research on human subjects. Informed consent was obtained from all participants prior to data collection. The patients/participants provided their written informed consent to participate in this study.

## Author contributions

AG was primarily responsible for the data analysis and manuscript writing. AG and JL were equally responsible for the conceptual and theoretical development of this manuscript and provided data for the study. All authors contributed to the article and approved the submitted version.

## Acknowledgments

We would like to thank the Department of Management, Marketing, and Information Systems at Sam Houston State for their assistance with open-access publication fees.

## Conflict of interest

The authors declare that the research was conducted in the absence of any commercial or financial relationships that could be construed as a potential conflict of interest.

## Publisher’s note

All claims expressed in this article are solely those of the authors and do not necessarily represent those of their affiliated organizations, or those of the publisher, the editors and the reviewers. Any product that may be evaluated in this article, or claim that may be made by its manufacturer, is not guaranteed or endorsed by the publisher.
